# General anesthesia technique and perception of quality of postoperative recovery in women undergoing cholecystectomy: A randomized, double-blinded clinical trial

**DOI:** 10.1371/journal.pone.0228805

**Published:** 2020-02-27

**Authors:** Daniel de Carli, José Fernando Amaral Meletti, Neri Edu Urnau Neto, Gabriel Martinez, André Luís Corrêa Kim, Rodrigo Pauperio Soares de Camargo

**Affiliations:** 1 Department of Anesthesiology, Jundiaí Medical School, Jundiaí, SP, Brazil; 2 Department of Obstetrics and Gynecology, Jundiaí Medical School, Jundiaí, SP, Brazil; Universita degli Studi di Roma La Sapienza, ITALY

## Abstract

**Background:**

The two most common general anesthesia techniques are total intravenous anesthesia (TIVA) and venous/inhalation balanced general anesthesia (BGA). It is unclear whether any of these two techniques affect patient perception of the quality of recovery. The aim of this randomized, double-blinded clinical trial was to assess the quality of postoperative recovery of women undergoing laparoscopic cholecystectomy under general anesthesia. We compared patients who received TIVA with those who received BGA. We also evaluated the factors that may decrease patient-perceived quality of postoperative recovery.

**Methods:**

We prospectively recruited 121 women aged 18–65 years who were scheduled for elective laparoscopic cholecystectomy due to cholelithiasis. These patients were randomized to receive TIVA (target-controlled infusion of propofol and remifentanil) or BGA (continuous remifentanil infusion and sevoflurane inhalation). To measure the quality of postanesthetic and postoperative recovery, we administered the Quality of Recovery-40 (QoR-40) questionnaire 24 hours after the patient awoke from anesthesia.

**Results:**

All 60 patients in the TIVA group responded to QoR-40 (median, 188 points; minimum 128; maximum 200). Sixty-one patients in the BGA group had a mean QoR-40 score of 186 points (median, 188 points; minimum 146; maximum 200). There was no significant difference in the QoR-40 score between the two groups (p = 0.577). The patients who presented postoperative nausea and vomiting (PONV) and pain had worse perception of the quality of postoperative recovery.

**Conclusions:**

Both TIVA and BGA had a similar effect on the perception of the quality of postoperative recovery in women undergoing elective laparoscopic cholecystectomy. PONV and pain may negatively affect patient perception of the quality of postoperative recovery.

## Introduction

Surgical and anesthetic procedures may initially negatively affect a patient’s quality of life, producing a sensation of discomfort, even in the absence of specific complications [1]. In addition, poor postoperative recovery may lead to increased hospital costs and decreased patient satisfaction [2, 3]. Therefore, anesthesiologists seek techniques that can provide high-quality recovery, minimize complications, and reduce the time to return to daily activities [4].

The two most commonly used general anesthesia techniques are total intravenous anesthesia (TIVA) and balanced general anesthesia (BGA), with intravenous anesthesia combined with inhalation anesthesia [5]. The use of TIVA has increased recently; this technique has been reported to provide a better experience and higher levels of patient satisfaction in outpatient and inpatient surgeries, possibly because of a lower incidence of pain, agitation, and postoperative nausea and vomiting (PONV) [6]. Studies on the quality of postanesthetic and postoperative recovery have mainly focused on measures such as recovery time, cardiorespiratory complications, pain, PONV, hospital length of stay, and other complications [7]. Considered in isolation, these factors may be insufficient to explain a patient’s perception of postoperative recovery following general anesthesia. However, patient-assessed quality of life is an important factor to be considered in this clinical setting.

The Quality of Recovery-40 (QoR-40) questionnaire measures the quality of recovery from anesthesia through five dimensions: physical comfort, physical independence, emotional state, psychological support, and pain [7, 8]. The validity, reliability, ease of use, responsiveness, and cross-cultural adaptation of QoR-40 to Portuguese have been confirmed previously [3, 9, 10]. This questionnaire has been used successfully in several clinical trials [11–13], but few studies have used it to test recovery following TIVA versus recovery following BGA [1, 14].

The aim of this clinical trial was to assess the quality of postoperative recovery of women undergoing elective laparoscopic cholecystectomy. We hypothesized that the perception of the quality of postoperative recovery 24 hours after waking from anesthesia would be better in women treated with total intravenous anesthesia than in women treated with balanced general anesthesia. We also evaluated the influence of pain, nausea and vomiting, and hypothermia on patient perception of the quality of postoperative recovery.

## Materials and methods

This randomized, double-blinded clinical trial complied with international standards of research ethics involving human subjects, based on Resolution 466/12 of the Brazilian Ministry of Health and the Declaration of Helsinki. This study was approved by the Research Ethics Committee of the Jundiaí Medical School (2.157.459) and by the Brazilian National System of Research Records Involving Human Beings ("Plataforma Brasil") (CAAE 69609417.5.0000.5412) and was registered prior to patient enrollment at the Brazilian Clinical Trials Registry (ReBEC) (RBR-7cwtgg, date of registration: 6/15/2017, URL: http://www.ensaiosclinicos.gov.br/rg/RBR-7cwtgg/). All patients provided written informed consent to participate in the study. Data were collected at a hospital of the Brazilian public health network located in a city in the state of São Paulo, Brazil, between 10/2/2017 and 10/31/2018.

Sample size was calculated based on the primary endpoint, which was the QoR-40 score, from a similar clinical trial that compared the quality of postoperative recovery on the first postoperative day in female patients undergoing thyroidectomy, between those who received TIVA and those who received BGA using desflurane [1]. In that clinical trial, the authors found a 13-point difference between the QoR-40 scores in the TIVA group (mean ± standard deviation [SD], 174 ± 17) and the BGA group (161 ± 22). Using these data and an SD of 22 points, we calculated the sample size as 60 patients per group in order to achieve a power of 90% and a type I error of 5%.

Women aged 18–65 years, who were scheduled to undergo an elective laparoscopic cholecystectomy due to cholelithiasis, with an American Society of Anesthesiologists [15] physical status I (normal healthy patient) or II (mild systemic disease), were included.

Exclusion criteria for the randomization process were as follows: patients who refused to participate in the study; patients taking any sedative, opioid, or sleep-inducing drugs; patients with a history of allergy to any drug used in the study; patients who had already undergone any surgery of the upper abdominal cavity; morbidly obese patients (defined by a body mass index [BMI] > 40 kg·m^-2^); patients who were pregnant or breastfeeding; carriers of pathologies that cause cognitive impairment (such as schizophrenia, oligophrenia, and depression).

Patients who, during surgery, required conversion to traditional (open) surgery, as decided by the surgeon, patients who presented complications requiring a new surgical intervention or transfer to the intensive care unit during hospitalization, and patients who chose to abandon the study were also excluded.

Patients were randomly assigned to the TIVA or BGA group by a researcher who was not responsible for performing anesthesia and did not collect the questionnaires. The randomization sequence was generated the morning of the surgery, using an internet-based random number generator available at www.random.org. Next, the researcher wrote the group to which the patient belonged in an opaque envelope, sealed it, and handed it to the anesthesiologist who performed the procedure according to the allocation declared in the envelope.

Due to considerable procedural differences between the two general anesthesia techniques, the anesthesiologist and the surgeons who attended the surgery were not blinded to patient allocation. However, both the patient and the researcher were unaware of the patient’s group until the questionnaire data were collected. Patients in the study did not receive preanesthetic medication. As soon as the patients entered the operating room, the following variables were monitored: peripheral oxygen saturation, plethysmography data, electrocardioscopy data, automatic non-invasive blood pressure measured every 5 minutes, oropharyngeal temperature, and bispectral index (BIS^®^)-measured anesthetic depth.

Sixty patients in the TIVA group received *target*-*controlled infusion* (TCI) with propofol and remifentanil using a commercial infusion pump and the Marsh (propofol) or Minto (remifentanil) pharmacological model. Anesthesia induction and maintenance steps aimed at a plasma concentration of the medication of 2–8 mcg·ml^-1^ for propofol and 2–8 ng·ml^-1^ for remifentanil, as guided by BIS^®^, which should remain between 45 and 60.

Sixty-one patients in the BGA group received a bolus administration of 1.5–2 mg·kg^-1^ of propofol and TCI of remifentanil (Minto) targeting a plasma concentration of 2–8 ng·ml^-1^. Anesthesia was maintained using 1.5–3% sevoflurane with adjunctive remifentanil infusion (2–8 ng·ml^-1^). The infusion rate of remifentanil and the expired concentration of sevoflurane were also guided by BIS^®^, which should remain between 45 and 60.

Neuromuscular blockade occurred with 0.6 mg·kg^-1^ rocuronium injected intravenously to facilitate intubation and pneumoperitoneum in all patients. During anesthesia, the patients were monitored with transcutaneous ulnar nerve stimulation and evaluated for the response of the adductor muscle of the thumb in the train of four stimuli (TOF). Tracheal intubation was performed in all patients after finding no muscular response, and a tracheal tube with a cuff was used, with an internal diameter ranging from 6.5 to 7.5 mm, calculated from the weight and BMI of the patient. The cuff pressure was maintained between 20 and 25 cm H_2_O. If the patient had a response greater than two stimuli in the assessment of neuromuscular blockade during surgery, a dose of 20% of the initial dose of rocuronium was administered.

Mechanical ventilation was maintained throughout the procedure with a tidal volume of 7 ml·kg^-1^, ventilatory frequency of 10–16 breaths per minute, and final positive expiratory pressure (PEEP) of 5–7 cm H_2_O, aiming for an end-tidal carbon dioxide (ETCO_2_) value between 35 and 45 mmHg. The mixture of gases with oxygen and ambient air gave the patients an inspired fraction of 50% oxygen. In both groups, mean blood pressure could vary within 20% of the pre-induction values. If necessary, hypotension was corrected with 5 mg of ephedrine, and hypertension was treated with an increased infusion of remifentanil.

Between 10 and 30 minutes before the end of surgery, patients in both groups received 8 mg ondansetron, 100 mg ketoprofen, 10 mg dexamethasone, and 100 mg tramadol intravenously. At the end of surgery, the surgical wound was infiltrated with 50% enantiomeric levobupivacaine hydrochloride at a concentration of 0.5% with vasoconstrictor, respecting the toxic dose of this anesthetic. After surgery was completed, all anesthetics were discontinued, and 0.05 mg·kg^-1^ of neostigmine, with 0.01 mg·kg^-1^ of intravenous atropine, was administered to reverse the possible residual effects of a neuromuscular blockade. The tracheal tube was removed as soon as consciousness was recovered and sufficient spontaneous breathing was confirmed. After verifying the oropharyngeal temperature and confirming stable vital signs and adequate breathing, the patients were transferred to the postanesthesia care unit (PACU).

Pain and PONV were assessed using a numerical rating scale (NRS) of 11 points on arrival at the PACU and every 10 minutes thereafter, until discharge to the hospital ward. When the pain score was equal to or greater than 4 points, the patient received 2 mg of morphine at 20-minute intervals until the pain score was less than 4 points. When the PONV score was greater than 3 points, the patient received a single 10-ml dose of the following mixture: 3 mg·ml^-1^ of dimenhydrinate, 5 mg·ml^-1^ of pyridoxine hydrochloride, 100 mg·ml^-1^ of glucose, and 100 mg·ml^-1^ of fructose.

After reaching a score equal to or greater than 9 in the modified Aldrete–Kroulik scale and a score of less than 4 in the evaluation of PONV and pain, the patients were discharged from the PACU and were referred to the hospital ward. During the stay in the hospital bed, the patients were medicated intravenously with 100 mg of ketoprofen every 12 hours as an analgesic medication. If they still had a pain score equal to or greater than 4, they received 100 mg of tramadol as rescue medication for pain. To prevent PONV, they received 8 mg of ondansetron every 8 hours.

The quality of postoperative functional recovery was measured at 24 hours upon awakening from anesthesia by the QoR-40 questionnaire in S1 and S2 Files, which evaluates five dimensions of recovery: physical comfort (12 items), emotional state (10 items), physical independence (4 items), psychological support (7 items), and pain (7 items). Each item was scored on a 5-point Likert scale. The questionnaire has two parts: In part A, the questions indicate positive aspects, and a higher score indicates a higher frequency of occurrence (1 point: at no time; 2 points: at times; 3 points: often; 4 points: most of the time; and 5 points: all the time). In part B, the questions indicate negative aspects, and a lower score indicates a higher frequency of occurrence (5 points: at no time; 4 points: at times; 3 points: often; 2 points: most of the time; and 1 point: all the time). The total score is the sum of all responses and can range from 40 (worse recovery quality) to 200 (better recovery quality).

In addition to QoR-40, patients and groups were compared on the basis of age, weight, height, occurrence of PONV and pain, hypothermia (defined as a temperature below 36° C at the end of surgery), duration of surgery, and length of stay in the PACU.

### Statistical analysis

After collection, quantitative data were entered into a database in Excel^®^ and transferred to and analyzed in SPSS^®^ 24. Categorical data and their frequencies were compared using Fisher’s exact test and Yates’ chi-square test. Normally distributed ordinal data (presented as the mean and SD) were compared using the Student’s t-test. Ordinal data and non-Gaussian continuous data (presented as the median and interval), which were not normally distributed as per the Kolmogorov–Smirnov test, were compared between groups using the Mann–Whitney test. A test power of 90% and a type I error of 5% were considered.

## Results

**Fig 1:**
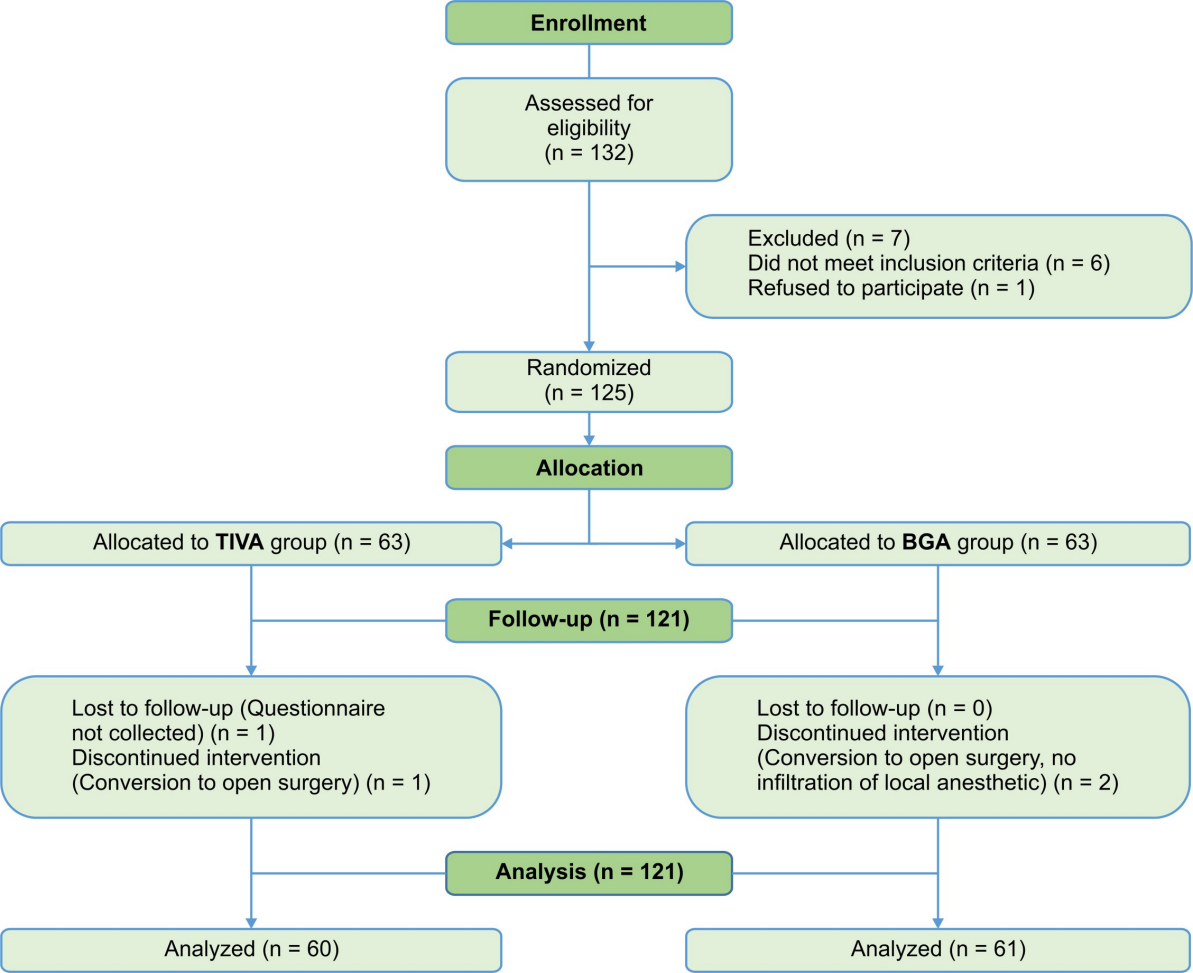
CONSORT flow diagram. Abbreviations: BGA, balanced general anesthesia; TIVA, total intravenous anesthesia.

A total of 132 patients were initially enrolled in this study. One of those patients refused to participate and 6 others did not meet the inclusion criteria; therefore, they were not considered for the randomization process. The remaining 125 patients signed the informed consent form and were randomized to TIVA or BGA; however, four patients were excluded during follow-up (two patients who required conversion for open surgery, one patient whose questionnaire was not collected, and one patient who did not receive local anesthetic into the surgical wound). Finally, the questionnaire data of 121 patients were collected and analyzed (Fig 1).

There were no significant differences between the two groups regarding age, BMI, duration of surgery, length of stay in the PACU, and temperature upon awakening from anesthesia Table 1.

**Table 1:** Demographic and anthropometric characteristics, length of stay in PACU, and temperature upon awakening from TIVA or BGA.

	Group	n	Mean	Standard deviation	Median	*p*
**Age** **(years)**	TIVA	60	43.78	13.24	44.0	0.941^a^
	BGA	61	43.95	11.43	42.0	
**BMI** **(kg·m**^**-2**^**)**	TIVA	60	27.89	4.10	27.5	0.695^a^
	BGA	61	27.56	5.04	27.4	
**Surgical time (minutes)**	TIVA	60	63.12	25.01	60.0	0.536^b^
	BGA	61	63.74	19.46	60.0	
**Length of stay in PACU (minutes)**	TIVA	60	78.33	36.85	65.0	0.818^b^
	BGA	61	75.15	31.79	65.0	
**Temperature upon awakening (°C)**	TIVA	60	36.04	0.50	36.0	0.236^b^
	BGA	61	35.87	0.52	36.0	

^*a*^Student’s t-test

^*b*^Mann–Whitney test.

Abbreviations: BGA, balanced general anesthesia; BMI, body mass index; n, number of cases; PACU, postanesthesia care unit; TIVA, total intravenous anesthesia

There were also no significant differences between the two groups regarding history of previous surgeries or pathologies, ASA physical status, presence of intraoperative complications, presence of PONV in the PACU, and need for rescue medication for pain (morphine) due to a pain score greater than 3 in the PACU Table 2.

**Table 2:** Physical status, intraoperative complications, pain, and PONV in PACU for patients who underwent TIVA or BGA.

		Group		Total n (%)	*p*
		TIVA n (%)	BGA n (%)		
**Previous surgeries**	Yes	39 (65.0)	33 (54.1)	72 (59.5)	0.300^b^
	No	21 (35.0)	28 (45.9)	49 (40.5)	
**ASA physical status**	I	25 (41.7)	28 (45.9)	53 (43.8)	0.775^b^
	II	35 (58.3)	33 (54.1)	68 (56.2)	
**Previous pathologies**	Yes	33 (55.0)	26 (42.6)	59 (48.8)	0.238^b^
	No	27 (45.0)	35 (57.4)	62 (51.2)	
**Intraoperative complications**	Yes	1 (1.7)	2 (3.3)	3 (2.5)	>0.999^c^
	No	59 (98.3)	59 (96.7)	118 (97.5)	
**PONV in PACU**	Yes	7 (11.7)	4 (6.6)	11 (9.1)	0.508^b^
	No	53 (88.3)	57 (93.4)	110 (90.9)	
**Need of morphine**^a^	Yes (≥1 doses)	26 (43.3)	18 (29.5)	44 (36.4)	0.164^b^
	No	34 (56.7)	43 (70.5)	77 (63.6)	

^*a*^Morphine was applied every 20 minutes if the patient reported a pain score > 3 on the numerical rating scale in the PACU

^*b*^Yates’ chi-square test

^*c*^Fisher’s exact test.

Abbreviations: ASA, American Society of Anesthesiology; BGA, balanced general anesthesia; n, number of cases; PACU, postanesthesia care unit; PONV, postoperative nausea and vomiting; TIVA, total intravenous anesthesia.

There were also no significant differences between the two groups regarding patient perception of the quality of postoperative recovery through the QoR-40 total score (p = 0.577), its individual domains (physical comfort, p = 0.619; emotional state, p = 0.447), physical independence (p = 0.129), psychological support (p = 0.111), pain (p = 0.337), and PONV at 24 hours upon awakening from anesthesia (p = 0.364) Table 3.

**Table 3:** QoR-40 scores and PONV at 24 hours after surgery for patients who underwent TIVA or BGA.

		Group	n	Median	Minimum score	Maximum score	*p*
**QoR-40**	**Total score**	TIVA	60	188	128	200	0.577^a^
		BGA	61	188	146	200	
		Total	121	188	128	200	
**QoR-40 domains**	**Physical comfort**	TIVA	60	56	33	60	0.619^a^
		BGA	61	57	38	60	
		Total	121	56	33	60	
	**Emotional state**	TIVA	60	48	37	50	0.447^a^
		BGA	61	48	31	50	
		Total	121	48	31	50	
	**Physical independence**	TIVA	60	18	4	20	0.129^a^
		BGA	61	19	9	20	
		Total	121	19	4	20	
	**Psychological support**	TIVA	60	35	7	35	0.111^a^
		BGA	61	35	30	35	
		Total	121	35	7	35	
	**Pain**	TIVA	60	33	13	35	0.337^a^
		BGA	61	32	14	35	
		Total	121	33	13	35	
	**PONV at 24 hours**^b^	TIVA	60	15	13	15	0.364^a^
		BGA	61	15	12	15	
		Total	121	15	12	15	

^*a*^Mann–Whitney test

^*b*^PONV at 24 hours was calculated as the sum of the answers related to the presence of nausea, vomiting, and vomiting without residues in the QoR-40.

Abbreviations: BGA, balanced general anesthesia; n, number of cases; PONV, postoperative nausea and vomiting; TIVA, total intravenous anesthesia; QoR-40, Quality of Recovery-40.

We analyzed the influence of moderate or severe pain in the PACU and in the first 24 hours postoperatively, PONV in the PACU and in the first 24 hours postoperatively, and hypothermia in patient-perceived quality of postoperative recovery Table 4.

**Table 4:** Influence of postoperative complications in patient-perceived quality of postoperative recovery, as assessed using QoR-40.

		n	Median	Minimum score	Maximum score	*p*
**Total**	QoR-40	121	188	128	200	
**Pain in PACU**	NRS ≤3	77	189	128	200	0.007^a^
	NRS >3	44	185	145	200	
**Pain in the first 24 hours**	NRS ≤3	66	189	128	200	0.009^a^
	NRS >3	55	186	145	200	
**PONV in PACU**	No	110	188	128	200	0.281^a^
	Yes	11	186	153	195	
**PONV in the first 24 hours**^b^	No	111	189	128	200	0.000^a^
	Yes	10	169	146	192	
**Temperature (°C)**^c^	≥36	65	190	160	200	0.159^a^
	<36	56	187	128	200	

^*a*^Mann–Whitney test

^***b***^PONV in the first 24 hours was calculated as the sum of the answers related to the presence of nausea, vomiting, and vomiting without residues in the QoR-40

^*c*^Temperature upon awakening from anesthesia.

Abbreviations: n, number of cases; NRS, numeric rating scale; PACU, postanesthesia care unit; PONV, postoperative nausea and vomiting; QoR-40, Quality of Recovery-40.

We found that patients who presented moderate or severe pain in the PACU (p = 0.007) or in the first 24 hours postoperatively (p = 0.009), as well as those who presented PONV in the first 24 hours postoperatively (p < 0.001) had a worse perception of the quality of postoperative recovery, as evaluated by the total QoR-40 score. Patients who presented PONV in the PACU (p = 0.281) or those who presented hypothermia upon awakening from anesthesia (p = 0.159) did not have a significantly lower QoR-40 score.

## Discussion

This clinical trial assessed and compared patient-perceived quality of postoperative recovery in women undergoing elective laparoscopic cholecystectomy, between those who received TIVA and those who received BGA. It also investigated the factors that may negatively affect this perception.

During the development and validation of the questionnaire (QoR-40), a lower average of the scores was attributed to the questionnaires answered by women compared to men [9]. Therefore, to avoid this type of bias, we chose to evaluate only women in this clinical trial.

There were no significant differences between the two groups regarding history of previous surgeries or pathologies, ASA physical status, presence of intraoperative complications, presence of PONV in the PACU, and need for rescue medication for pain, which demonstrates adequate group randomization and concealment. Factors such as age, obesity, previous surgeries, and associated pathologies are associated with complications during the postoperative period and may affect the quality of postoperative recovery [8, 13, 16–19]. The standard use of analgesic and antiemetic medications in the intra and postoperative period may explain the lack of difference in PONV and pain between the two groups.

The overall QoR-40 score was high (mean, 185.4; median, 188), denoting a good evaluation of the quality of recovery by the patients. There were no significant differences between the two groups regarding patient-perceived quality of postoperative recovery, possibly because there were also no significant differences in undesirable side effects, such as PONV, pain, and hypothermia.

In this study, all patients received 10 mg of parenteral dexamethasone prior to the onset of anesthesia. The perioperative use of dexamethasone reduces the incidence of pain, nausea, and fatigue and shortens the period of stay in the hospital after laparoscopic cholecystectomy, leading to better evaluation of the quality of recovery by the patients [4]. In addition, surgical wound infiltration with levobupivacaine has been performed for all patients, which, together with postoperative analgesia, may increase the quality of recovery [20, 21]. The use of these drugs and techniques associated with analgesics and antiemetics administrated parenterally (tramadol, ketoprofen, and ondansetron) may help understand the lack of differences in the study outcomes between the two groups as well as the high QoR-40 scores.

A randomized clinical trial reported better perception of the quality of postoperative recovery among women undergoing thyroidectomy who received TIVA with remifentanil and propofol compared to those who received BGA with propofol, remifentanil, and desflurane [1]. Differences between the aforementioned study and the present study regarding the anesthetic techniques used, including the inhalant agent (desflurane vs. sevoflurane), the analgesic and antiemetic medications used (ramosetron and ketorolac vs. ondansetron, ketoprofen, and tramadol), the surgery performed (thyroidectomy vs. cholecystectomy), and the characteristics of the study populations, may explain the discrepancies between the results.

A meta-analysis also related better perception of the quality of postoperative recovery in the TIVA group, which was attributed to lower frequency of PONV, agitation, and postoperative pain [6]. However, the methodological heterogeneity of the studies may explain the difference between the results obtained in that meta-analysis and our study.

Another clinical trial evaluated the quality of postoperative recovery in 110 men and women undergoing otorhinolaryngologic surgery with either TIVA (remifentanil and propofol) or BGA (remifentanil, propofol, and sevoflurane) with a population sample from a region close to the population of this clinical trial [14]. As in the present study, the authors found no differences between the two groups regarding quality of recovery, PONV, pain, and hypothermia. Although the study was carried out in patients of both sexes and the type of surgery was different, the socioeconomic similarity of the population sample in both studies may justify the similarity between the results.

An important aspect that may help understand the high QoR-40 scores in both groups was the monitoring and maintenance of a deep intraoperative neuromuscular blockade. Laparoscopic surgeries under low abdominal pressure are associated with a lower incidence of postoperative pain and referred pain in the shoulder, 24 hours after surgery [22]. In addition, monitoring of neuromuscular relaxation prevents residual blockage upon awakening, which may cause adverse respiratory events, such as hypoxia, upper airway obstruction, and a prolonged stay in the PACU, that may decrease patient-perceived quality of recovery [15, 23, 24].

In this study, we found that the presence of moderate or intense pain in the PACU or in the first 24 hours significantly decreased patient-perceived quality of postoperative recovery. The same phenomenon occurred when patients reported PONV in the first 24 hours, but not in the PACU. As for hypothermia upon awakening, no significant decrease in QoR-40 scores was observed. As in our study, postoperative pain [13, 16–18] and PONV [13, 17, 18] have been associated with lower QoR-40 scores in other studies.

Although hypothermia is related to complications such as increased cardiac morbidity, infection at the surgical site, residual effects of anesthetic drugs, increased PACU stay, coagulopathies, hormonal changes, and tremors [19], these possible complications did not lead to a decrease in QoR-40 scores in patients undergoing orthopedic lower limb surgeries in an observational study [13], similar to our present findings.

During this clinical trial, difficulties were encountered in measuring the quality of recovery, due to the subjective aspect of the tested domains, which may be influenced by emotional fragility, presence of side effects, fasting, and residual effects of drugs that may alter patient cognition [25]. The comparison between the results obtained herein and those from the literature is also hampered by the possibility of interpretation bias due to sociocultural differences of the studied populations, which may affect the perception of the quality of recovery. This complexity of factors was the main limiting factor in this study.

Despite these limitations, the increasing concern about patient perception of the quality of health care requires anesthesiologists to constantly seek anesthesia techniques that provide better quality of recovery. In this study, even though we did not observe superiority of either one of the two general anesthesia techniques, we found that achieving better patient-perceived quality of postoperative recovery may involve minimizing the side effects, such as PONV and pain.

## Conclusion

The choice of general anesthesia technique did not influence the perception of the quality of postoperative recovery in women undergoing elective laparoscopic cholecystectomy, but PONV and pain should be given special attention in this population.

## Supporting information

S1 File Questionnaire QoR-40. English version.
(DOCX)

S2 File Questionnaire used in the research. Includes the QoR-40 Portuguese (BRA) version questionnaire.
(DOCX)

S3 File CONSORT checklist. (DOC)

S4 File Approved research protocol. Portuguese (BRA) version.
(PDF)

S5 File Approved research protocol. English version.
(DOCX)
