# Supplementary material for: General anesthesia technique and perception of quality of postoperative recovery in women undergoing cholecystectomy: A randomized, double-blinded clinical trial
Source: PLoS One. 2020 Feb 27;15(2):e0228805. doi: 10.1371/journal.pone.0228805 (PMC7046219; doi:10.1371/journal.pone.0228805)
Supplement: S1 File — English version. (DOCX) [file pone.0228805.s001.docx]

# S1. QoR-40 questionnaire in the original language (English version):

**Patient Survey (QoR–40)**

## Part A:

How have you been feeling in the last 24 hours?

(1 to 5, where : 1 = very poor and 5 = excellent)

For example: If you have been able to breathe easily all of the time, you should indicate this by circling the response 5 = all of the time as shown below:

| Able to breathe easily | **None of the time** | **Some of the time** | **Usually** | **Most of the time** | **All of the time** |
| --- | --- | --- | --- | --- | --- |
|  | 1 | 2 | 3 | 4 | 5 |

| **Confort** | **None of the time** | **Some of the time** | **Usually** | **Most of the time** | **All of the time** |
| --- | --- | --- | --- | --- | --- |
| Able to breathe easily | **1** | **2** | **3** | **4** | **5** |
| Have had a good sleep | **1** | **2** | **3** | **4** | **5** |
| Been able to enjoy food | **1** | **2** | **3** | **4** | **5** |
| Feel rested | **1** | **2** | **3** | **4** | **5** |

| **Emotions** | **None of the time** | **Some of the time** | **Usually** | **Most of the time** | **All of the time** |
| --- | --- | --- | --- | --- | --- |
| Having a feeling of general well-being | **1** | **2** | **3** | **4** | **5** |
| Feeling in control | **1** | **2** | **3** | **4** | **5** |
| Feeling comfortable | **1** | **2** | **3** | **4** | **5** |

| **Physical Independence** | **None of the time** | **Some of the time** | **Usually** | **Most of the time** | **All of the time** |
| --- | --- | --- | --- | --- | --- |
| Have normal speech | **1** | **2** | **3** | **4** | **5** |
| Able to wash, brush teeth or shave | **1** | **2** | **3** | **4** | **5** |
| Able to look after your own appearance | **1** | **2** | **3** | **4** | **5** |
| Able to return to work or usual home activities | **1** | **2** | **3** | **4** | **5** |

| **Patient Support** | **None of the time** | **Some of the time** | **Usually** | **Most of the time** | **All of the time** |
| --- | --- | --- | --- | --- | --- |
| Able to communicate with hospital staff (when in hospital) | **1** | **2** | **3** | **4** | **5** |
| Able to communicate with family or friends | **1** | **2** | **3** | **4** | **5** |
| Getting support from hospital doctors (when in hospital) | **1** | **2** | **3** | **4** | **5** |
| Getting support from hospital nurses (when in hospital) | **1** | **2** | **3** | **4** | **5** |
| Having support from family or friends | **1** | **2** | **3** | **4** | **5** |
| Able to write | **1** | **2** | **3** | **4** | **5** |
| Able to understand instructions and advice | **1** | **2** | **3** | **4** | **5** |

## PART B:

Have you had any of the following in the last 24 hours?

(5 to 1, where: 5 = excellent and 1 = very poor)

| **Confort** | **None of the time** | **Some of the time** | **Usually** | **Most of the time** | **All of the time** |
| --- | --- | --- | --- | --- | --- |
| Nausea | **5** | **4** | **3** | **2** | **1** |
| Vomiting | **5** | **4** | **3** | **2** | **1** |
| Dry-retching | **5** | **4** | **3** | **2** | **1** |
| Feeling restless | **5** | **4** | **3** | **2** | **1** |
| Shaking or twitching | **5** | **4** | **3** | **2** | **1** |
| Shivering | **5** | **4** | **3** | **2** | **1** |
| Feeling too cold | **5** | **4** | **3** | **2** | **1** |
| Feeling dizzy | **5** | **4** | **3** | **2** | **1** |

| **Emotions** | **None of the time** | **Some of the time** | **Usually** | **Most of the time** | **All of the time** |
| --- | --- | --- | --- | --- | --- |
| Had bad dreams | **5** | **4** | **3** | **2** | **1** |
| Feeling anxious | **5** | **4** | **3** | **2** | **1** |
| Feeling angry | **5** | **4** | **3** | **2** | **1** |
| Feeling depressed | **5** | **4** | **3** | **2** | **1** |
| Feeling alone | **5** | **4** | **3** | **2** | **1** |
| Had difficulty falling asleep | **5** | **4** | **3** | **2** | **1** |
| Feeling confused | **5** | **4** | **3** | **2** | **1** |

| **Pain** | **None of the time** | **Some of the time** | **Usually** | **Most of the time** | **All of the time** |
| --- | --- | --- | --- | --- | --- |
| Moderate pain | **5** | **4** | **3** | **2** | **1** |
| Severe pain | **5** | **4** | **3** | **2** | **1** |
| Headache | **5** | **4** | **3** | **2** | **1** |
| Muscle pains | **5** | **4** | **3** | **2** | **1** |
| Backache | **5** | **4** | **3** | **2** | **1** |
| Sore throat | **5** | **4** | **3** | **2** | **1** |
| Sore mouth | **5** | **4** | **3** | **2** | **1** |

Thank you for your assistance.
