# Supplementary material for: General anesthesia technique and perception of quality of postoperative recovery in women undergoing cholecystectomy: A randomized, double-blinded clinical trial
Source: PLoS One. 2020 Feb 27;15(2):e0228805. doi: 10.1371/journal.pone.0228805 (PMC7046219; doi:10.1371/journal.pone.0228805)
Supplement: S2 File — Includes the QoR-40 Portuguese (BRA) version questionnaire. (DOCX) [file pone.0228805.s002.docx]

# Questionnaire used for the research, which includes QoR-40 in the translated and cross-culturally adapted version for the Portuguese language (BRA):

***Qualidade de recuperação pós-operatória em mulheres submetidas a colecistectomias: ensaio clínico randomizado e duplamente encoberto***

**Esta parte deve ser respondida pelo pesquisador**

Caso n^o^ : _______

Nome:________________________________________________________________________________

Idade:_____anos Data_____/_____/________

Prontuário:_________________________________

**Colar etiqueta de Identificação**

Peso: ____kg Altura ______cm

ASA______ Tempo cirúrgico: ______ min.

Temperatura ao final da cirurgia_______^o^C

Histórico de patologias anteriores: ___________________________________________________________________

Antecedentes cirúrgicos: __________________________________________________________________________

Apresentou náuseas ou vômitos na sala de cirurgia ou SRPA? ⃝Não ⃝Sim

Terapêutica utilizada na SRPA: _____________ _____mg, _____________ _____mg, _______________ _____mg

Apresentou dor na sala de cirurgia ou SRPA (EAV≥4)? ⃝Não ⃝Sim

Terapêutica utilizada na SRPA: _____________ _____mg, _____________ _____mg, _______________ _____mg

Complicações cirúrgicas ou anestésicas nessa internação? ⃝Não ⃝Sim Quais____________________________________________________________________________________

Tempo de permanência na SRPA_____________________minutos.

**Esta parte deve ser respondida pela paciente**

Qual o nível de dor que sentiu nas últimas 24h, na escala de 0 a 10? A extremidade esquerda (nota 0) representa “nenhuma dor” e a extremidade direita (nota 10) representa “a pior dor imaginável”.


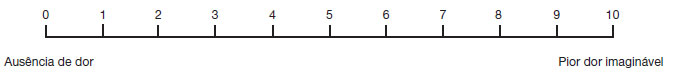


Qual seu nível geral de satisfação com a **anestesia** que recebeu, na escala de 0 a 10? A extremidade esquerda (nota 0) representa “muito insatisfeita com a anestesia” e a extremidade direita (nota 10) representa “muito satisfeita com a anestesia”


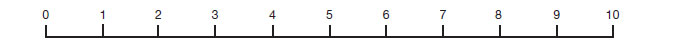
**Qor-40**

Muito Satisfeita

Muito Insatisfeita

Responda às questões conforme o exemplo a seguir: Como você tem se sentido nas últimas 24 horas?

**Se você sente que é capaz de respirar facilmente todo o tempo, você deve circular a resposta 5=todo o tempo, como demonstrado acima.**

Em nenhum Alguns Frequente Maior parte Tempo todo

momento momentos do tempo

Capaz de respirar facilmente 1 2 3 4 5

Parte A: (circule respostas de 1 a 5, onde 1=muito ruim e 5=excelente)

Como você tem se sentido nas últimas 24 horas?

| **Conforto Físico** | **Em nenhum momento** | **Alguns momentos** | **Frequente** | **Maior parte do tempo** | **Tempo todo** |
| --- | --- | --- | --- | --- | --- |
| Capaz de respirar facilmente | **1** | **2** | **3** | **4** | **5** |
| Conseguiu dormir bem | **1** | **2** | **3** | **4** | **5** |
| Capaz de saborear o que come | **1** | **2** | **3** | **4** | **5** |
| Sente-se descansado(a) | **1** | **2** | **3** | **4** | **5** |

| **Emoções** | **Em nenhum momento** | **Alguns momentos** | **Frequente** | **Maior parte do tempo** | **Tempo todo** |
| --- | --- | --- | --- | --- | --- |
| Sensação agradável de bem estar | **1** | **2** | **3** | **4** | **5** |
| Sente-se controlado(a) | **1** | **2** | **3** | **4** | **5** |
| Sente-se confortável | **1** | **2** | **3** | **4** | **5** |

| **Independência física** | **Em nenhum momento** | **Alguns momentos** | **Frequente** | **Maior parte do tempo** | **Tempo todo** |
| --- | --- | --- | --- | --- | --- |
| Consegue falar | **1** | **2** | **3** | **4** | **5** |
| Capaz de tomar banho, escovar dentes ou se barbear | **1** | **2** | **3** | **4** | **5** |
| Capaz de cuidar de sua aparência | **1** | **2** | **3** | **4** | **5** |
| Sente-se capaz de voltar ao trabalho ou tarefas domésticas | **1** | **2** | **3** | **4** | **5** |

| **Apoio** | **Em nenhum momento** | **Alguns momentos** | **Frequente** | **Maior parte do tempo** | **Tempo todo** |
| --- | --- | --- | --- | --- | --- |
| Capaz de comunicar com a equipe de profissionais | **1** | **2** | **3** | **4** | **5** |
| Capaz de se comunicar com família ou amigos | **1** | **2** | **3** | **4** | **5** |
| Sente apoio dos médicos | **1** | **2** | **3** | **4** | **5** |
| Sente apoio da equipe de enfermagem | **1** | **2** | **3** | **4** | **5** |
| Sente apoio da família ou amigos | **1** | **2** | **3** | **4** | **5** |
| Capaz de escrever | **1** | **2** | **3** | **4** | **5** |
| Capaz de entender instruções e  avisos | **1** | **2** | **3** | **4** | **5** |

PARTE B

Você apresentou qualquer sintoma relacionado abaixo, nas últimas 24 horas?

(respostas 5 a 1, onde 5=excelente e 1=muito ruim)

| **Conforto Físico** | **Em nenhum momento** | **Alguns momentos** | **Frequente** | **Maior parte do tempo** | **Tempo todo** |
| --- | --- | --- | --- | --- | --- |
| Náusea | **5** | **4** | **3** | **2** | **1** |
| Vômitos | **5** | **4** | **3** | **2** | **1** |
| Vômito sem conteúdo | **5** | **4** | **3** | **2** | **1** |
| Sente inquietude | **5** | **4** | **3** | **2** | **1** |
| Espasmos musculares | **5** | **4** | **3** | **2** | **1** |
| Tremores | **5** | **4** | **3** | **2** | **1** |
| Sente muito frio | **5** | **4** | **3** | **2** | **1** |
| Sente tontura | **5** | **4** | **3** | **2** | **1** |

| **Emoções** | **Em nenhum momento** | **Alguns momentos** | **Frequente** | **Maior parte do tempo** | **Tempo todo** |
| --- | --- | --- | --- | --- | --- |
| Teve pesadelos | **5** | **4** | **3** | **2** | **1** |
| Sente-se ansioso(a) | **5** | **4** | **3** | **2** | **1** |
| Sente-se bravo(a) | **5** | **4** | **3** | **2** | **1** |
| Sente-se deprimido(a) | **5** | **4** | **3** | **2** | **1** |
| Sente-se solitário(a) | **5** | **4** | **3** | **2** | **1** |
| Dificuldade para começar a dormir | **5** | **4** | **3** | **2** | **1** |
| Sente-se confuso(a) | **5** | **4** | **3** | **2** | **1** |

| **Dor** | **Em nenhum momento** | **Alguns momentos** | **Frequente** | **Maior parte do tempo** | **Tempo todo** |
| --- | --- | --- | --- | --- | --- |
| Dor moderada | **5** | **4** | **3** | **2** | **1** |
| Dor severa | **5** | **4** | **3** | **2** | **1** |
| Dor de cabeça | **5** | **4** | **3** | **2** | **1** |
| Dor em músculos | **5** | **4** | **3** | **2** | **1** |
| Dor nas costas | **5** | **4** | **3** | **2** | **1** |
| Dor de garganta | **5** | **4** | **3** | **2** | **1** |
| Machucados na Boca | **5** | **4** | **3** | **2** | **1** |

Por favor, confira se todas as perguntas foram respondidas. Obrigado pela colaboração!

**Ao final das respostas da paciente, o pesquisador deverá verificar se todas as questões foram assinaladas e anotar o grupo ao qual a paciente pertenceu.**

**⃝AGVT (TIVA) ⃝AGVI (Balanceada)**
