# Supplementary material for: General anesthesia technique and perception of quality of postoperative recovery in women undergoing cholecystectomy: A randomized, double-blinded clinical trial
Source: PLoS One. 2020 Feb 27;15(2):e0228805. doi: 10.1371/journal.pone.0228805 (PMC7046219; doi:10.1371/journal.pone.0228805)
Supplement: S4 File — Portuguese (BRA) version. (PDF) [file pone.0228805.s004.pdf]

PARECER CONSUBSTANCIADO DO CEP

DADOS DO PROJETO DE PESQUISA

Título da Pesquisa: Qualidade de recuperação pós-operatória em mulheres submetidas a colecistectomias: Ensaio clínico randomizado e duplamente encoberto.

Pesquisador: Daniel de Carli

Área Temática:

Versão: 1

CAAE: 69609417.5.0000.5412

Instituição Proponente: Faculdade de Medicina de Jundiaí

Patrocinador Principal: Financiamento Próprio

DADOS DO PARECER

Número do Parecer: 2.157.455

Apresentação do Projeto:

Informações fornecidas pelo Pesquisador no arquivo "PB_INFORMAÇÕES_BÁSICAS_DO_PROJETO_907530.pdf":

"Resumo:

Duas técnicas comuns de anestesia geral são a anestesia venosa total e anestesia balanceada venosa e inalatória. Ainda não está claro se alguma dessas técnicas afeta a percepção do paciente de sua qualidade de recuperação. Este ensaio clínico visa avaliar recuperação de mulheres submetidas a colecistectomias laparoscópicas sob anestesia geral, comparando as que receberão anestesia geral venosa total com aquelas que receberão anestesia balanceada. Oitenta mulheres com idade entre 18 e 65 anos serão prospectivamente recrutadas e randomizadas para os grupos AGVT (infusão controlada por alvo de propofol e remifentanil) ou Balanceada (com infusão contínua de remifentanil e inalação de sevoflurano). O questionário QoR-40 será administrado para avaliar a qualidade da recuperação pós-anestésica e pós-operatória vinte e quatro horas após a administração da anestesia. A incidência de náuseas ou vômitos, dor e hipotermia na unidade de recuperação pós-anestésica e durante a internação hospitalar também serão avaliadas."

Endereço: Rua Francisco Telles, 250
Bairro: Vila Arens **CEP:** 13.202-550
UF: SP **Município:** JUNDIAI
Telefone: (11)4587-1095 **Fax:** (11)4587-1095 **E-mail:** cep@fmj.br

Objetivo da Pesquisa:

Informações fornecidas pelo Pesquisador no arquivo "PB_INFORMAÇÕES_BÁSICAS_DO_PROJETO_907530.pdf":

"Hipótese:

Existe diferença entre as duas técnicas anestésicas mais comumente empregadas na anestesia geral quanto à percepção da qualidade de recuperação pós-operatória em colecistectomias laparoscópicas em mulheres.

Objetivo Primário:

Avaliar a qualidade de recuperação pós-operatória de pacientes submetidas a colecistectomias laparoscópicas eletivas, comparando as pacientes que receberão a AGVT com infusão alvo controlada de propofol e remifentanil, e as pacientes que receberão AGVI com remifentanil e sevoflurano.

Objetivo Secundário:

Avaliar a influência da dor, náuseas e vômitos e hipotermia na percepção da qualidade de recuperação pós operatória das pacientes submetidas a colecistectomias laparoscópicas eletivas."

Avaliação dos Riscos e Benefícios:

Informações fornecidas pelo Pesquisador no arquivo "PB_INFORMAÇÕES_BÁSICAS_DO_PROJETO_907530.pdf":

"Riscos:

Os riscos relacionados à pesquisa são os mesmos daqueles que estão presentes diante de uma anestesia geral para cirurgias eletivas para colecistectomias laparoscópicas na população estudada. Não existem medicamentos ou técnicas experimentais. As técnicas e medicamentos utilizados são bem fundamentados em literatura científica e fazem parte do cotidiano da equipe de anestesiologia do Hospital Regional de Jundiaí. Ocorrerá, apenas, a comparação entre duas técnicas consagradas (AGVT e AGVI) a partir da percepção da paciente.

Benefícios:

Após o seu encerramento, esse ensaio clínico ajudará a melhorar a qualidade do atendimento às mulheres submetidas a colecistectomias laparoscópicas, através da determinação (ou não) de uma

técnica anestésica em que avaliem que tiveram uma melhor sensação de bem estar."

Comentários e Considerações sobre a Pesquisa:

Informações fornecidas pelo Pesquisador no arquivo "PB_INFORMAÇÕES_BÁSICAS_DO_PROJETO_907530.pdf":

"Metodologia Proposta:

Este ensaio clínico randomizado duplamente encoberto deverá ser aprovado comitê de ética em pesquisa da Faculdade de Medicina de Jundiaí e pela plataforma Brasil. O termo de consentimento informado deverá ser assinado por todas as participantes. Cada paciente será aleatoriamente designada para o grupo AGVT ou AGVI. A randomização será feita através de um gerador de números aleatórios baseado na web e disponível em www.random.org. O anestesiologista que assistirá a cirurgia saberá o grupo ao qual pertence a paciente, porém, a paciente e o pesquisador não terão conhecimento quanto à identidade de grupo. Após monitorizadas, as pacientes alocadas ao grupo AGVT receberão uma anestesia com infusão alvo controlada (TCI) de propofol e remifentanil utilizando os modelos farmacológicos de Schnider para o propofol e de Minto para o remifentanil. A indução e manutenção anestésicas objetivarão uma concentração plasmática da medicação de 2 a 8 mcg.ml-1 para o propofol e 2 a 8 ng.ml-1 para o remifentanil, determinadas a partir da avaliação do BIS, que deverá permanecer entre 45 e 60, e orientará a diminuição ou aumento da concentração das medicações. No grupo AGVI, as pacientes receberão a administração em bolus de 1,5 a 2 mg.kg-1 de propofol e 1 a 2 mcg.kg-1 de remifentanil (durante 3 minutos), a anestesia será mantida utilizando 1,5 a 3% de sevoflurano com infusão adjuvante de 0,05 a 0,2 mcg.kg-1.min-1 de remifentanil. A velocidade de infusão do remifentanil e a concentração expirada de sevoflurano também serão reguladas pela avaliação do BIS que deverá permanecer entre 45 e 60. O bloqueio neuromuscular com 0,6 mg.kg-1 de rocurônio será injetado por via endovenosa para facilitar a intubação e o pneumoperitôneo em todas as pacientes. Durante a anestesia, as pacientes serão monitorizadas com o estímulo transcutâneo do nervo ulnar e avaliadas quanto a resposta do músculo adutor do polegar na sequência de quatro estímulos. A intubação traqueal será realizada em todas as pacientes. Entre 10 e 30 minutos antes do final da cirurgia as pacientes de ambos os grupos receberão 8mg de ondasentrona, 100mg de cetoprofeno, 2 g de dipirona, 10 mg dexametasona e 100mg de tramadol. Ao final da cirurgia, a ferida cirúrgica será infiltrada com levobupivacaína a 0,5% c/v. Ao término da cirurgia, todos os anestésicos serão descontinuados e, após a constatação da presença de sinais vitais estáveis e respiração espontânea os pacientes serão extubados e transferidos para a sala de recuperação

Endereço: Rua Francisco Telles, 250
Bairro: Vila Arens **CEP:** 13.202-550
UF: SP **Município:** JUNDIAI
Telefone: (11)4587-1095 **Fax:** (11)4587-1095 **E-mail:** cep@fmj.br

pós-anestésica.A qualidade da recuperação funcional pós-operatória será avaliada utilizando o questionário QoR-40, que avalia cinco dimensões da recuperação: conforto físico (12 itens), estado emocional (9 itens), independência física (5 itens), apoio psicológico (7 itens) e dor (7 itens). A pontuação em cada item será feita por uma escala Likert de cinco pontos, sendo que valores elevados correspondem à melhor resposta (1 ponto: em nenhum momento, 2 pontos: em alguns momentos, 3 pontos: frequentemente, 4 pontos: na maior parte do tempo e 5 pontos: o tempo todo).O escore será estabelecido pela somatória das respostas em cada item, e varia de 40 (má qualidade de recuperação) a 200 (melhor qualidade de recuperação). A QoR-40 será administrada 24 horas após a realização da cirurgia.Além da Qor-40, as pacientes serão avaliadas e os grupos serão comparados quanto a idade, peso, altura, duração anestésica, ocorrência de dor, náuseas, vômitos e hipotermia pós-operatórios.O cálculo do tamanho da amostra foi baseado a partir de um ensaio clínico semelhante, que avaliou o Qor-40 no primeiro dia de pós-operatório de mulheres submetidas a tireoidectomias, comparando-se AGVT com AGVI com desflurano. Este ensaio concluiu que 34 indivíduos por grupo seriam suficientes para se atingir uma potência de 90% com um erro de tipo 1 de 0,05. A fim de permitir uma taxa de abandono de de até 20%, serão alocados para esse ensaio clínico um total de 80 pacientes."

Os critérios de inclusão e exclusão e a segurança envolvendo as participantes estão muito claros. A metodologia é adequada e o estudo é aceitável.

Considerações sobre os Termos de apresentação obrigatória:

A Folha de Rosto foi apresentada devidamente assinada pelo Pesquisador e pelo Responsável pela Instituição Proponente.

O TCLE tem linguagem acessível, informa claramente o objetivo, os riscos, benefícios e segurança envolvendo o participante, além de detalhar todos os procedimentos do estudo. Consta que é direito dos participantes abandonar o estudo a qualquer momento sem qualquer ônus e sem a necessidade de prestar esclarecimentos ao Pesquisador. O TCLE contempla, portanto, todos os itens da Resolução nº 466/12 do CNS.

Recomendações:

Não há.

Conclusões ou Pendências e Lista de Inadequações:

O presente Projeto de Pesquisa encontra-se em conformidade com a Resolução nº 466/12 do CNS

e foi submetido para aprovação na plenária de 05/07/2017 do Comitê de Ética em Pesquisa da Faculdade de Medicina de Jundiaí.

Considerações Finais a critério do CEP:

O Comitê de Ética em Pesquisa da Faculdade de Medicina de Jundiaí ressalta que é responsabilidade do Pesquisador enviar relatórios semestrais e relatório de eventos adversos, caso esses venham a ocorrer, assim como relatório final com os resultados da pesquisa, para finalização do protocolo.

Este parecer foi elaborado baseado nos documentos abaixo relacionados:

Tipo Documento	Arquivo	Postagem	Autor	Situação
Informações Básicas do Projeto	PB_INFORMAÇÕES_BÁSICAS_DO_PROJETO_907530.pdf	30/05/2017 22:01:11		Aceito
Folha de Rosto	Folha_de_rosto_Qualidade.pdf	30/05/2017 22:00:23	Daniel de Carli	Aceito
Declaração de Instituição e Infraestrutura	Declaracao_de_instituicao_coparticipante.jpg	30/05/2017 21:50:50	Daniel de Carli	Aceito
Projeto Detalhado / Brochura Investigador	Projeto_Detalhado_Qualidade_em_Colecistectomias.docx	28/05/2017 08:12:11	Daniel de Carli	Aceito
Outros	Questionario.docx	06/05/2017 21:21:59	Daniel de Carli	Aceito
TCLE / Termos de Assentimento / Justificativa de Ausência	TCLEQor40.docx	06/05/2017 21:19:17	Daniel de Carli	Aceito

Situação do Parecer:

Aprovado

Necessita Apreciação da CONEP:

Não

JUNDIAI, 05 de Julho de 2017

Assinado por:

João Baptista Opitz Neto

(Coordenador)
