# Supplementary material for: General anesthesia technique and perception of quality of postoperative recovery in women undergoing cholecystectomy: A randomized, double-blinded clinical trial
Source: PLoS One. 2020 Feb 27;15(2):e0228805. doi: 10.1371/journal.pone.0228805 (PMC7046219; doi:10.1371/journal.pone.0228805)
Supplement: S5 File — English version. (DOCX) [file pone.0228805.s005.docx]

CONSUMPTED OPINION OF THE REC

## RESEARCH PROJECT DATA

Research Title:  "General anesthesia technique and perception of quality of postoperative recovery in women undergoing cholecystectomy: a randomized, double-blinded clinical trial"

Version: 1

CAAE: 69609417.5.0000.5412

Author: Daniel de Carli

Proponent Institution: Jundiaí Medical School

Primary Sponsor: Own Financing

OPINION DATA

Opinion Number: 2.157.455

Project presentation:

Summary:

Two common general anesthesia techniques are total intravenous anesthesia and intravenous and inhaled balanced anesthesia. It is not yet clear whether any of these techniques affect the patient's perception of their quality of recovery. This clinical trial aims to evaluate the recovery of women undergoing laparoscopic cholecystectomies under general anesthesia, comparing those who will receive total intravenous general anesthesia with those who will receive balanced anesthesia. Eighty women aged 18 to 65 years will be prospectively recruited and randomized to either TIVA (propofol and remifentanil target-controlled infusion) or Balanced (with continuous remifentanil infusion and sevoflurane inhalation) groups. The QoR-40 questionnaire will be administered to assess the quality of postoperative and postoperative recovery 24 hours after anesthesia administration. The incidence of nausea or vomiting, pain and hypothermia in the post-anesthetic recovery unit and during hospitalization will also be assessed. "

Research Purpose:

"Hypothesis:

There is a difference between the two anesthetic techniques most commonly used in general anesthesia regarding the perception of postoperative recovery quality in laparoscopic cholecystectomies in women.

Primary Objective:

To evaluate the quality of postoperative recovery in patients undergoing cholecystectomies

laparoscopic studies comparing patients receiving controlled target infusion of propofol and remifentanil, and patients receiving remifentanil and sevoflurane TIVA.

Secondary Objective:

To evaluate the influence of pain, nausea and vomiting and hypothermia on the perception of postoperative recovery quality of patients undergoing elective laparoscopic cholecystectomies.

Risk and Benefit Assessment:

Risks:

The risks related to the research are the same as those present with general anesthesia for elective surgeries for laparoscopic cholecystectomies in the study population. There are no drugs or experimental techniques. The techniques and drugs used are well based on scientific literature and are part of the daily life of the anesthesiology team of the Jundiaí Regional Hospital. There will only be a comparison between two established techniques (TIVA and BGA) from the patient's perception.

Benefits:

Upon completion, this clinical trial will help improve the quality of care for women undergoing laparoscopic cholecystectomies by determining (or not) an anesthetic technique in which they feel they have a better sense of well-being. "

Research Comments and Considerations:

Proposed Methodology:

This double-blind randomized clinical trial must be approved by the Research Ethics Committee of the Jundiaí Medical School and by the Brazil platform (National Etic Committee). The informed consent form must be signed by all participants. Each patient will be randomly assigned to the TIVA or BGA group. Randomization will be done through a web-based random number generator available at www.random.org. The anesthesiologist attending the surgery will know the group to which the patient belongs, but the patient and the researcher will not be aware of the group identity. After monitoring, patients assigned to the TIVA group will receive propofol and remifentanil target-controlled infusion anesthesia (TCI) using the Marsh pharmacological models for propofol and Minto for remifentanil. Anesthetic induction and maintenance will target a plasma drug concentration of 2 to 8 mcg.ml-1 for propofol and 2 to 8 ng.ml-1 for remifentanil, determined from the BIS assessment, which should remain between 45 and 60, and will guide the decrease or increase in drug concentration. In the BGA group, patients will receive bolus administration of 1.5 to 2 mg.kg-1 propofol and 1 to 2 mcg.kg-1 remifentanil (for 3 minutes), anesthesia will be maintained using 1.5 to 2 mg.kg-1. Sevoflurane 3% with adjuvant infusion of 0.05 to 0.2 mcg.kg-1.min-1 remifentanil. Remifentanil infusion rate and expired sevoflurane concentration will also be regulated by BIS evaluation which should remain between 45 and 60. Neuromuscular blockade with 0.6 mg.kg-1 rocuronium will be injected intravenously to facilitate intubation. and pneumoperitoneum in all patients. During anesthesia, patients will be monitored with transcutaneous ulnar nerve stimulation and evaluated for the response of the adductor pollicis muscle following four stimuli. Tracheal intubation will be performed in all patients. Between 10 and 30 minutes before the end of surgery, patients in both groups will receive 8mg of ondansetron, 100mg of ketoprofen, 10mg dexamethasone and 100mg of tramadol. At the end of the surgery, the surgical wound will be infiltrated with 0.5% levobupivacaine with epinephrine. At the end of surgery, all anesthetics will be discontinued and, after the presence of stable vital signs and spontaneous breathing, patients will be extubated and transferred to the post-anesthetic recovery room (PACU). The quality of postoperative functional recovery will be evaluated using the QoR-40 questionnaire, which assesses five dimensions of recovery: physical comfort (12 items), emotional state (9 items), physical independence (5 items), psychological support (7 items) and pain (7 items). Scores on each item will be scored on a five-point Likert scale, with high values ​​corresponding to the best answer (1 point: at no time, 2 points: sometimes 3 points: often 4 points: most of the time). time and 5 points: all the time). The score will be established by the sum of the answers in each item, and ranges from 40 (poor quality of recovery) to 200 (best quality of recovery). QoR-40 will be administered 24 hours after surgery. In addition to Qor-40, patients will be evaluated and groups will be compared for age, weight, height, anesthetic duration, occurrence of pain, nausea, vomiting and post-operative hypothermia. Sample size calculation was based on a similar clinical trial evaluating Qor-40 on the first postoperative day of women undergoing thyroidectomies, comparing AGVT with AGVI with desflurane. This trial concluded that 34 subjects per group would be sufficient to achieve 90% potency with a type 1 error of 0.05. In order to allow a dropout rate of up to 20%, a total of 80 patients will be allocated to this trial. "

The inclusion and exclusion criteria and the safety involving the participants are very clear. The methodology is adequate and the study is acceptable.

Compulsory Terms Considerations:

The Cover Sheet was duly signed by the Researcher and the Head of the Proposing Institution.

The ICF has accessible language, clearly informs the participant's purpose, risks, benefits and safety, and details all study procedures. It is stated that participants are entitled to leave the study at any time without charge and without the need to provide clarification to the Researcher. Therefore, the informed consent includes all items of Resolution No. 466/12 of the CNS.

Recommendations: none

Conclusions or Pending Issues and List of Inadequacies:

This Research Project is in compliance with CNS Resolution No. 466/12 and was submitted for approval at the plenary session of 07/05/2017 of the Research Ethics Committee of the Jundiaí Medical School.

Final Considerations at CEP's discretion:

The Research Ethics Committee of the Jundiaí Medical School emphasizes that it is the responsibility of the Researcher to send semiannual reports and adverse event reports, should they occur, as well as a final report with the research results, to finalize the protocol.

This opinion is based on the following documents:

Type Document File Post Author Situation

Project Basic Information

PB_BASIC_INFORMATION_PROJECT_907530.pdf 30/05/2017 22:01:11 Accepted

Cover Sheet

Folha_de_rosto_Qualidade.pdf 30/05/2017 22:00:23 Daniel de Carli Accepted

Institution and Infrastructure Statement

Declaration_of_institution_coparticipant.jpg 30/05/2017 21:50:50 Daniel de Carli Accepted

Detailed Project / Brochure / Researcher

Project_Detailed_Quality_on_Colecystectomies.docx 28/05/2017 8:12:11 Daniel de Carli Accepted

Others

Questionnaire.docx 05/05/2017 21:21:59 Daniel de Carli Accepted

ICF / Terms of Consent / Absence Justification

TCLEQor40.docx 06/05/2017 21:19:17 Daniel de Carli Accepted

Opinion Status:

Approved

Needs CONEP Appraisal:

Not

Email: cep@fmj.br

Address: 250, Francisco Telles Street

Neighborhood: Vila Arens

ZIP Code: 13.202-550

Phone: 55 (11) 4587-1095

State: SP

Municipality: JUNDIAÍ

Fax: 55(11) 4587-1095

JUNDIAI, July 05, 2017

**João Baptista Opitz Neto**

(Coordinator)
